# Efficacy of titanium brush, 915 nm diode laser, citric acid for eradication of *Staphylococcus aureus* from implant surfaces

**DOI:** 10.1186/s12903-021-01997-z

**Published:** 2021-12-07

**Authors:** Mohammad Reza Karimi, Behshad Farkhondemehr, Motahare Ghaeni Najafi, Ardavan Etemadi, Nasim Chiniforush

**Affiliations:** 1 Department of Periodontics, Faculty of Dentistry, Tehran Medical Sciences, Islamic Azad University, Tehran, Iran; 2 Faculty of Dentistry, Tehran Medical Sciences, Islamic Azad University, Tehran, Iran; 3 Laser Research Center of Dentistry, Dentistry Research Institute, Tehran University of Medical Sciences, Tehran, Iran

**Keywords:** Peri-implantitis, Titanium brush, Diode laser, Citric acid

## Abstract

**Background:**

This study aimed to assess the efficacy of titanium brush, 915 nm diode laser, citric acid and the combination of latter two with titanium brush for decontamination of SLA surface mini-implants.

**Methods:**

Seventy-five mini-implants contaminated with *Staphylococcus aureus* (*S. aureus*) were randomly divided into five experimental groups (n = 12) of titanium brush (TiB), laser, citric acid (CA), brush-laser, and brush-acid, positive [n = 12; chlorhexidine mouthwash (CHX)] and negative [n = 2; phosphate buffered saline (PBS)] control groups and one no-treatment group (n = 1). After counting the colony forming units (CFUs), data were analyzed using the Kruskal–Wallis and Dunn post-hoc tests.

**Results:**

Regardless of the no-treatment and negative control groups, maximum and minimum CFUs were noted in the titanium brush and positive control groups. After CHX, minimum CFUs were noted in brush-acid group followed by brush-laser, laser, and acid groups. Generally, the Kruskal–Wallis test revealed a significant difference between the groups regarding the colony count (P < 0.001). Dunn post-hoc test showed that the difference between the titanium brush and acid-brush group was significant (P < 0.001) while the differences between the brush and laser groups with the brush-laser group were not significant (P > 0.077).

**Conclusions:**

Combined use of titanium brush and citric acid yielded superior results compared to other groups in reduction of *S. aureus* on implant surface.

## Background

With the increasing use of dental implants in dental rehabilitation of edentulous patients, the rate of related complications is also rising [1, 2]. Peri-implantitis is the most common complication that refers to inflammation of the implant surrounding structures, causing their destruction. Peri-implantitis occurs in 10% of implants and 20% of patients within 5 to 10 years after implant placement [3, 4]. If continues, the inflammatory mediators invade the junctional epithelium and bone marrow space and eventually cause bone destruction and compromise the implant stability and function. The pathogenesis of peri-implantitis involves colonization of oral flora microorganisms and periodontal pathogens on the implant surface [5–7]. Thus, its treatment should include infection control, decontamination of implant surface, regeneration of the lost tissue, and plaque control programs [4]. The conventional treatment methods for peri-implantitis include the use of curettes and sonic and ultrasonic scaling instruments [8]. Nonetheless, complete elimination of bacterial biofilm from the rough implant surface is not predictably feasible [9–11]. Moreover, these instruments can damage the implant surface and impair osteoblast adhesion and reosseointegration to implant [12].

Titanium brush is a mechanical instrument, which can be used for debridement of implant surfaces as an alternative to other mechanical instruments. Many studies have reported the optimal plaque removal efficacy of titanium brush with minimal surface modifications [13, 14]. However, more favorable results can be obtained by the combination use of titanium brush and chemical agents [14]. Evidence shows that citric acid has maximum decontamination efficacy among other chemical agents [15]. However, some studies have mentioned its irritability and toxicity for periodontal tissues when used in high concentrations [15, 16].

Laser application is a relatively novel modality that can be effectively used as an adjunct to the mechanical methods for treatment of peri-implantitis [4]. The efficacy of decontamination by different laser types such as diode lasers depends on their thermal effect and subsequent denaturation of proteins and cell lysis [17]. Diode lasers are able to decontaminate implants with no surface damage [18–20]. Thus, this study aimed to compare the efficacy of titanium brush, 915 nm diode laser, citric acid and the combination of latter two with titanium brush for decontamination of mini-implant surfaces contaminated with *Staphylococcus aureus.*

## Methods

**Fig. 1:**
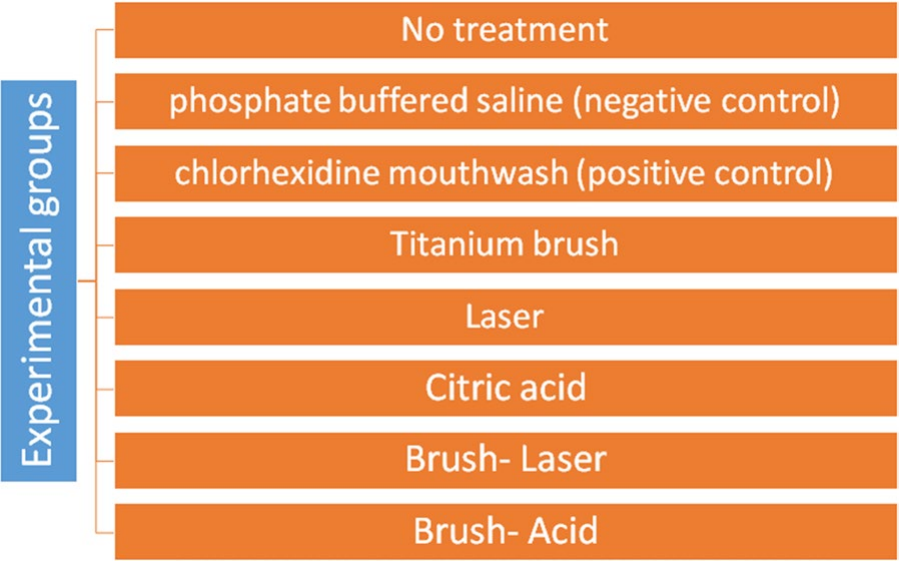
Flowchart of study groups

**Fig. 2:**
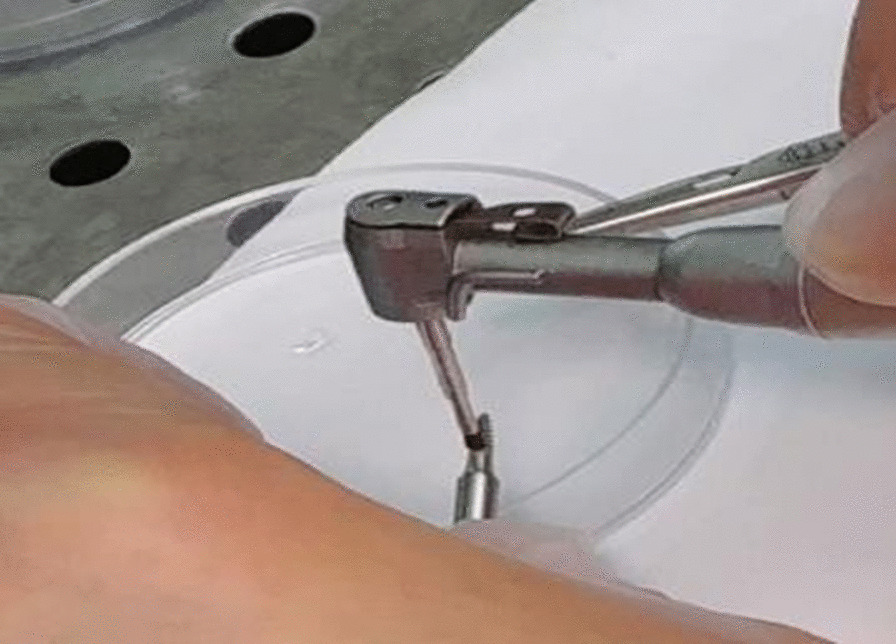
Application of titanium brush on the mini-implant surface

Seventy-five ball type titanium mini-implants with SLA (sandblasted and acid-etched) surface (Dentium, Seoul, South Korea) measuring 2 × 6 mm were bought from company distributor for this in-vitro study. They were randomly divided into five experimental, two control and one no-treatment groups (Fig. 1). The experimental and positive control groups included 12 mini-implants each. The negative control group included two mini-implants and the no-treatment group included one mini-implant [21]. A culture medium containing *S. aureus* (IBRC-M 10690) was obtained. For the purpose of contamination, each sterile mini-implant was immersed in 1 mL of *S. aureus* solution [mean concentration of 1 × 10^8^ colony forming units (CFUs) per milliliter] [19]. The plates were then incubated at 37 °C and 5% CO2 for 72 h. Eventually, prior to decontamination, the mini-implants were dipped into sterile phosphate buffered saline (PBS; Gibco, Darmstadt, Germany) three times to eliminate unattached (planktonic) bacteria. Then, the two mini-implants in the negative control group were immersed in sterile PBS for 60 s. In the positive control group, 12 mini-implants were immersed in 0.2% v/w chlorhexidine (CHX) mouthwash for 60 s and then immersed in sterile PBS three times to eliminate excess CHX [13]. In the titanium brush group, a titanium brush (with bristles of 3 mm in length and 1 mm in diameter) was attached to a low-speed (20:1) contra-angle hand- piece (NSK, Tokyo, Japan) operating at 150 rpm with clockwise/counter-clockwise rotation, while the hand-piece was connected to a surgical micro-motor (220 V; NSK, Tokyo, Japan). 12 mini-implants in this group were debrided with the titanium brush for 120 s which was positioned perpendicular to the implant surface and was used in one single direction for each mini-implant (Fig. 2). The applied pressure to the hand-piece remained constant at 50 N/cm^2^ [13]. Each mini-implant was held by means of forceps from its crown. The debridement process was performed by the same operator.

**Fig. 3:**
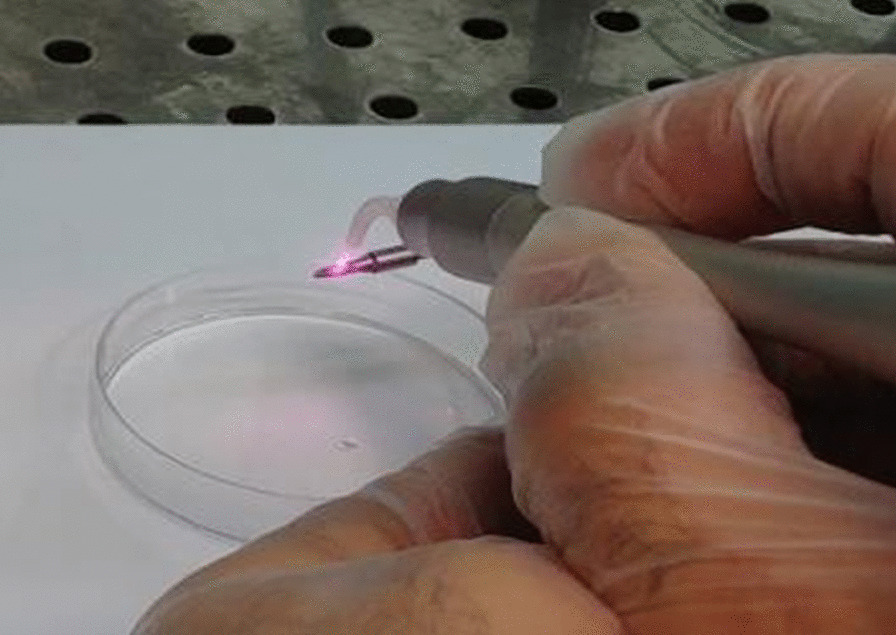
Irradiation of diode laser on the mini-implant surface

For decontamination of each mini-implant in the citric acid group, an autoclave-sterilized swab was dipped in 40% citric acid (pH = 1) and gently rubbed on the surface for 2 min uniformly from the crown to the apex. To eliminate excess citric acid, each mini-implant was dipped into PBS with a forward–backward movement for three rounds, each round for 10 times [16]. The same operator applied citric acid on 12 mini-implants in this group. In the titanium brush- citric acid group, each of the 12 mini-implants was first brushed for 120 s and then citric acid was applied for 2 min as explained earlier. In the laser group, each of the 12 mini-implants underwent diode laser irradiation with 915 nm wavelength (88Dent, Milan, Italy) and 1 W power in continuous-wave mode with a 300 µm non-initiated fiber tip for 60 s at 1 mm distance [22] (Fig. 3). To ensure complete laser irradiation of all surfaces, each mini-implant was attached to a low-speed hand-piece operating at 20 rpm and during rotation, the laser hand-piece also moved along the longitudinal axis of the mini-implant [19]. The same operator irradiated all mini-implants. In the titanium brush-diode laser group, titanium brush was first used for 12 mini-implants for 120 s and then laser was irradiated for 60 s with methods as explained earlier. After decontamination, each mini-implant was immersed in 1 mL of brain heart infusion (BHI) broth (Merck, Darmstadt, Germany) and then vortexed by maximum intensity (up and down) for 1 min to detach the bacteria from the mini-implant surface. The obtained solution was serially diluted by sterile saline using the drop plate method. For colony counting, 50 µL of each dilution was cultured on BHI broth agar and incubated under microaerophilic conditions for 48 h. The CFUs were counted by a single blind examiner and reported separately for each sample [23].

## Results

**Fig. 4:**
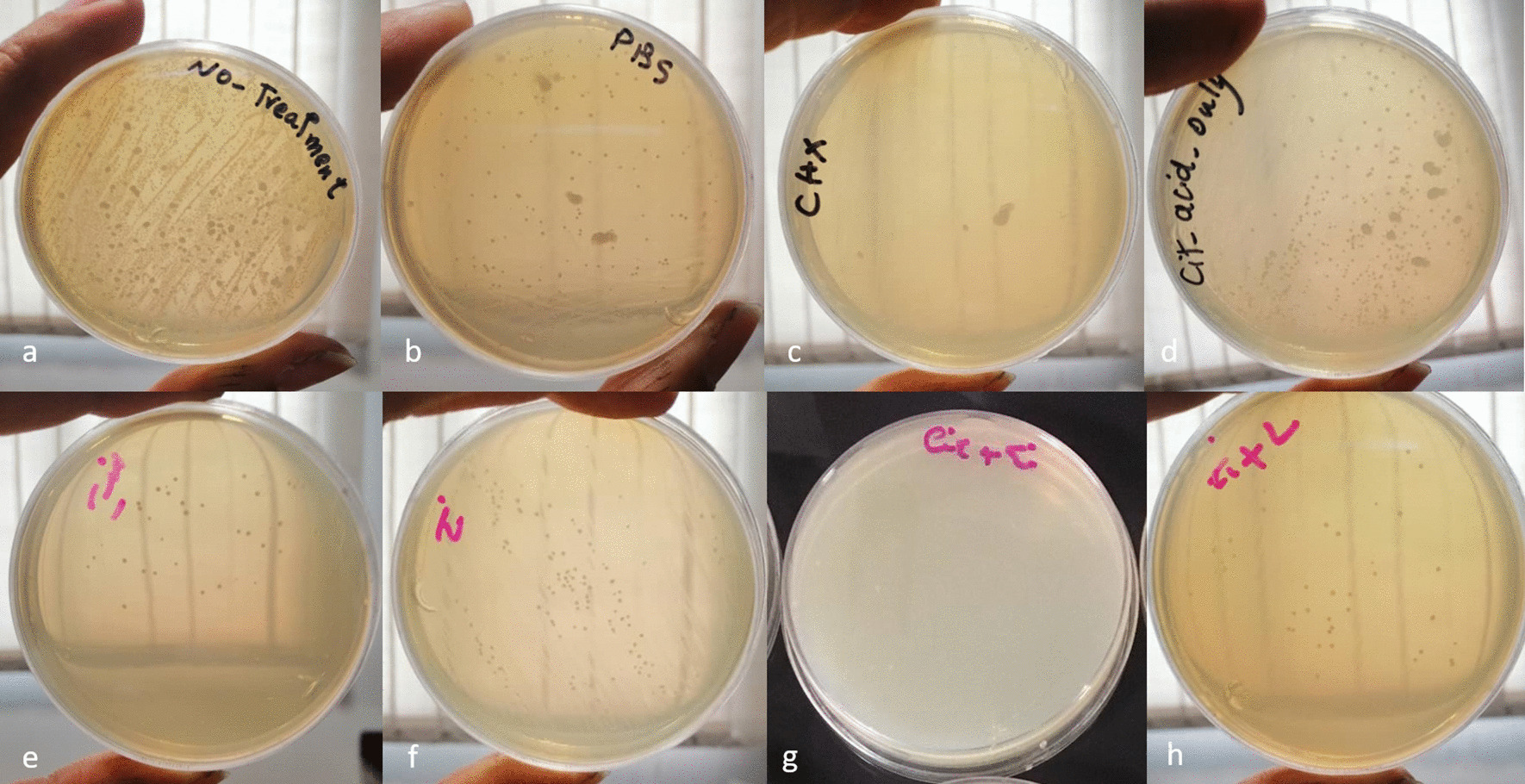
Plate of **a** no treatment, **b** PBS, **c** CHX, **d** citric acid, **e** laser, **f** titanium brush, **g** titanium brush-citric acid, **h** titanium brush-laser

The mean and standard deviation of experimental groups were presented in Table 1. As shown, maximum and minimum CFUs (regardless of the negative control and no-treatment groups) were found in the titanium brush and CHX groups. After CHX, minimum CFUs were noted in brush-acid, brush-laser, laser, and acid groups, in an ascending order (Fig. 4).

**Table 1:** Comparison of the mean and standard deviation of *S. aureus* CFUs in the six groups (all groups except for the negative control and no-treatment groups; n = 12)

Groups	Minimum	Maximum	Mean	Std. deviation
TiB + CA	200	1300	608.3333	352.80263
CA	100	12,500	4041.6667	4789.84880
TiB + laser	600	4500	1908.3333	1151.64573
Laser	500	9000	2825.0000	2253.93595
TiB	2500	8000	4566.6667	1953.70666
CHX (positive control)	0	1500	241.6667	439.95523
PBS (negative control)	500,000	1,000,000	647,500	204,618.547

*TiB* titanium brush, *CT* citric acid, *CHX* chlorhexidine

The Kruskal–Wallis test showed a significant difference between the study groups (P < 0.001). The Dunn post-hoc test was applied for pairwise comparisons of the groups, which revealed significant differences between the CHX group and each of the titanium brush, citric acid, laser, and brush-laser groups (P ≤ 0.007). Also, a significant difference was noted between the titanium brush and brush-acid groups (P < 0.001). Pairwise comparisons of other groups (Table 2) revealed no significant difference (P ≥ 0.077) for instance the brush-acid and brush-laser (P = 0.405), and brush-acid and laser (P = 0.077) groups. Table 2 shows pairwise comparisons precisely.

**Table 2:** P values for pairwise comparisons of the six groups regarding *S. aureus* CFUs

	TiB	CA	Laser	TiB + Laser	TiB + CA	CHX
TiB	–	> 0.999	> 0.999	0.536	** < 0.001**	** < 0.001**
CA		–	> 0.999	> 0.999	0.326	**0.005**
Laser			–	> 0.999	0.077	**0.001**
TiB + Laser				–	0.405	**0.007**
TiB + CA					–	> 0.999
CHX						–

Bold numbers show significant difference

*TiB* titanium brush, *CA* ciatric acid, *CHX* chlorhexidine

## Discussion

The current results showed that there was no significant difference in colony count between the CHX positive control group and brush-acid group (P > 0.999), which means that these two groups had almost similar efficacy for the reduction of *S. aureus* colonies. No significant difference was noted between each of the brush and laser groups, and the brush-laser group; this finding indicated that combined use of laser and titanium brush adds no advantage, and the use of each modality alone can yield the same result as their combined use. However, a significant difference existed between the brush-acid group and the titanium brush group, which indicated that the combined use of titanium brush and acid yielded significantly superior results (P < 0.001). In 2016, Saffarpour et al., compared the antimicrobial effects of photodynamic therapy, Er:YAG laser and 2% CHX in the elimination of *Aggregatibacter actinomycetemcomitans* biofilm from SLA implant surfaces CHX group showed the lowest and the control group showed the highest colony count. Their results were in agreement with ours, which may be due to the adoption of similar methodology in saline and CHX groups [21].

Carral et al. (2016) in treatment of peri-implantitis in dogs found that the use of titanium brush can be an acceptable modality for the treatment of peri-implantitis [24]. Also in 2018, Vigano et al., found that the application of titanium brush can effectively help in marginal bone regain in peri-implantits management in dogs. After five months, the dogs were reevaluated for marginal bone level radiographically, and the results revealed 0.6 mm bone gain in both groups with no significant difference between the saline and titanium brush groups. In other words, both methods effectively managed peri-implantitis in short-term [25].

In contrast, our results and those of some other studies [13, 21] indicated that saline had minimum efficacy for decontamination of implant surfaces, and its efficacy was much lower than that of titanium brush. This controversy in the results may be due to different application methods of saline (application of a gauze soaked in saline and then rinsing with saline and repeating this procedure for ten times). Also, duration, speed, torque and direction of titanium brush usage and duration of the saline application were not mentioned in their study. Toma et al. (2019) compared the efficacy of three mechanical modalities for the treatment of peri- implantitis in a randomized clinical trial on 47 patients. These modalities included the use of plastic curettes, air- abrasion and titanium brush. The patients were evaluated regarding plaque index, gingival index, bone loss and periodontal pocket depth at three time points namely initiation of treatment, three months after surgery, and six months after surgery. They reported that these mechanical methods improved the periodontal parameters in a short time. The reduction of periodontal pocket depth and gingival index in the titanium brush group was greater than that in other groups. They reported that titanium brush and glycine air-polishing were more effective than the other modality; however, the percentage of treatment success was still low; thus, they should be used in combination with antibacterial agents or antibiotics to yield more favorable results [26]. Despite the reportedly optimal efficacy of titanium brush in some studies, some others stated that it yields more favorable results when used in combination with other modalities. In this regard, Widodo et al. (2015) assessed the efficacy of different methods for decontamination of titanium surfaces contaminated with *S. aureus* and showed that the use of titanium brush along with a non-mechanical modality is the most efficient method for reduction of *S. aureus* colony count on the polished and SLA implant surfaces, yielding superior results compared with the application of titanium brush or photodynamic therapy alone [13]. De Tapia et al. (2019) assessed the efficacy of combined use of titanium brush, ultrasonic scaler and 3% hydrogen peroxide during regenerative surgery in a clinical trial on 30 patients. After one year of follow-up, clinical and radiographic examinations revealed that using titanium brush in regenerative surgery for treatment of peri-implantitis was highly effective and significantly decreased bone loss and pocket depth [27]. In line with our findings, Htet et al. (2016) performed a clinical study on dogs with peri-implantitis and concluded that the combined use of titanium burs and citric acid, compared with the use of titanium burs alone, resulted in better bone-implant contact and yielded superior results compared with Er: YAG laser in vertical bone height regeneration. This study was the first to use round titanium burs operating at 800 rpm under copious saline irrigation for 2 min; whereas, in previous studies, diamond burs were used to smoothen the implant surface and threads (implantoplasty). However, the 1-mm diameter of titanium burs prevented their complete access to the space between the threads. Thus, for complete debridement, use of a chemical agent along with the mechanical factor was imperative. Radiographic and histologic analyses yielded two important findings: (I) The combined use of citric acid and titanium bur yielded the best results, (II) Citric acid was able to eliminate the smear layer. They used cotton pellets dipped in 40% citric acid and gently rubbed them on the implant surfaces for 2 min, which was similar to our methodology [28]. Dalago et al. (2019) in their clinical trial, highlighted the significance of using citric acid as the most efficient chemical agent following mechanical debridement on 27 patients. They used 50% citric acid gel (pH = 1) for 3 min [29]. Concentration, form and duration of citric acid application in their study were different from ours; however, the results showed that citric acid, either alone or in combination with other modalities such as subepithelial connective tissue grafts or implantoplasty, could be effective in the treatment of peri-implantitis and increase the survival rate of implants.

Regarding the diode laser, Valente et al. (2017) assessed 130 implants placed in bone blocks with standardized vertical defects and contaminated with *Streptococcus sanguinis*. Next, they decontaminated the implants using 810 and 980 nm diode lasers. They showed that the application of diode laser at these wavelengths significantly decreased the contamination. Similar to our study, they used 1 W laser for 60 s at a 3 mm distance. A noteworthy issue is that this specific wattage was selected after the results of pilot studies indicated that 0.6 and 0.8 wattages were not sufficient and powers higher than 1 W caused histological changes in bone [22]. Goncalvez et al. (2010) contaminated the machined, titanium oxide sandblasted, and SLA implant surfaces with *Porphyromonas gingivalis* and *Enterococcus faecalis* and then decontaminated them using 980 nm diode and Nd: YAG lasers with two different powers. Reduction in colony count (in variable percentages) was noted in all groups. The reduction in colony count was 100% on SLA implant surfaces contaminated with *Porphyromonas gingivalis* and decontaminated with diode laser at both powers. However, the reduction in colony count was 50% on SLA implant surfaces contaminated with *Enterococcus faecalis* and decontaminated with 2.5 W diode laser, and 100% in the same group when 3 W laser was used. Differences between their results and ours may be due to the use of different bacterial species, also different wavelengths and powers of diode laser. Also, lasers were used for 5 min in their study [22]. In contrast, a review study by Lin et al. (2016) on 11 articles, regarding the application of laser for treatment of peri-implantitis and peri-implant mucositis showed that using laser in combination with surgical or non-surgical treatment modalities had low efficacy for decreasing the probing depth and clinical attachment loss. However, application of laser combined with non-surgical treatment modalities decreased bleeding on probing in short-term [30]. This can be due to the fact that this review article evaluated clinical studies and did not include in vitro studies. Also, the diode lasers used in these studies had different wavelengths than ours.

In this study, PBS and CHX showed minimum and maximum decontamination efficacy, respectively. However, despite the ease of use and availability of CHX, we tried to find alternative methods since previous studies revealed that CHX compromises the biocompatibility of titanium surfaces and is therefore not recommended for treatment of peri-implantitis [31].

It should be noted that this study had an in vitro design, and clinical studies are required to confirm its results prior to their generalization to the clinical setting.

## Conclusion

Our findings indicated that the application of titanium brush alone was not highly effective in the treatment of peri-implantitis, but it should be used in combination with some other modalities. Its combination with citric acid yielded better results than its combined use with 915 nm diode laser in this study. Moreover, the application of citric acid has a lower cost and enhances the biocompatibility of titanium surfaces, and is therefore effective and safe for the treatment of peri-implantitis.

## Data Availability

The datasets used and/or analyzed during the current study are available from the corresponding author on reasonable request.

## Abbreviations

*S.** aureus*: *Staphylococcus aureus*
TiB: Titanium brush
CA: Citric acid
CHX: Chlorhexidine
PBS: Phosphate buffered saline
CFUs: Colony forming units
